# Greater Ethanol-Induced Locomotor Activation in DBA/2J versus C57BL/6J Mice Is Not Predicted by Presynaptic Striatal Dopamine Dynamics

**DOI:** 10.1371/journal.pone.0083852

**Published:** 2013-12-12

**Authors:** Jamie H. Rose, Erin S. Calipari, Tiffany A. Mathews, Sara R. Jones

**Affiliations:** 1 Department of Physiology and Pharmacology, Wake Forest School of Medicine, Winston-Salem, North Carolina, United States of America; 2 Department of Chemistry, Wayne State University, Detroit, Michigan, United States of America; University of Chicago, United States of America

## Abstract

A large body of research has aimed to determine the neurochemical factors driving differential sensitivity to ethanol between individuals in an attempt to find predictors of ethanol abuse vulnerability. Here we find that the locomotor activating effects of ethanol are markedly greater in DBA/2J compared to C57BL/6J mice, although it is unclear as to what neurochemical differences between strains mediate this behavior. Dopamine elevations in the nucleus accumbens and caudate-putamen regulate locomotor behavior for most drugs, including ethanol; thus, we aimed to determine if differences in these regions predict strain differences in ethanol-induced locomotor activity. Previous studies suggest that ethanol interacts with the dopamine transporter, potentially mediating its locomotor activating effects; however, we found that ethanol had no effects on dopamine uptake in either strain. *Ex vivo* voltammetry allows for the determination of ethanol effects on presynaptic dopamine terminals, independent of drug-induced changes in firing rates of afferent inputs from either dopamine neurons or other neurotransmitter systems. However, differences in striatal dopamine dynamics did not predict the locomotor-activating effects of ethanol, since the inhibitory effects of ethanol on dopamine release were similar between strains. There were differences in presynaptic dopamine function between strains, with faster dopamine clearance in the caudate-putamen of DBA/2J mice; however, it is unclear how this difference relates to locomotor behavior. Because of the role of the dopamine system in reinforcement and reward learning, differences in dopamine signaling between the strains could have implications for addiction-related behaviors that extend beyond ethanol effects in the striatum.

## Introduction

 DBA/2J (DBA) and C57BL/6J (C57) mice are two inbred strains that show disparate phenotypes with respect to ethanol preference, drinking, and reward, among many other ethanol-mediated behaviors [1-6]. The strains’ differential responses to ethanol exposure are often thought to model behaviors associated with alcohol abuse vulnerability, abuse and dependence. For example, C57 mice demonstrate high levels of voluntary ethanol intake, but little conditioned place preference (CPP) to ethanol, a measure of reward, while DBA mice voluntarily consume little ethanol but exhibit robust CPP for an ethanol-paired environment [1-3]. Because DBA and C57 mice demonstrate differential responses to ethanol-mediated behaviors, these strains have become valuable tools for examining the individual differences that predict ethanol abuse vulnerability.

 In addition to the differences in drinking behavior and ethanol reward between DBA and C57 mice, DBA mice are more sensitive to the locomotor-activating effects of ethanol [7,8], although the neurochemical differences that are driving these behavioral disparities are unclear. Many studies have demonstrated that increases in dopamine in the ventral (nucleus accumbens, NAc) and dorsal (caudate-putamen, CPu) striatum mediate locomotor responses to drugs of abuse, including ethanol [9-12]. Although it has been shown that ethanol significantly increases striatal dopamine levels, the precise mechanisms by which ethanol enhances locomotor activity is unclear. Striatal dopamine increases have been attributed to a number of factors including increases in ventral tegmental area (VTA) dopamine cell firing [13-15] and ethanol effects directly on striatal dopamine terminals [16-18]. Previous work has demonstrated that the locomotor-enhancing effects of stimulant drugs such as cocaine and amphetamine are due to their specific actions on presynaptic dopamine terminals, where they inhibit the dopamine transporter (DAT) to cause increases in synaptic dopamine levels [9,19,20]. It has been argued previously that ethanol has direct actions on the DAT [16-18], and differences between ethanol-DAT interactions could underlie strain differences in the locomotor-activating properties of ethanol. Here, we aimed to determine if ethanol has direct effects on dopamine terminals that could in part explain the disparities in ethanol-induced locomotion between strains.

 The primary focus of the current research was to assess ethanol-induced locomotor activity in DBA and C57 mice, and whether or not this activity was mediated by altered dopamine dynamics at the level of striatal terminals. DBA mice exhibited much greater locomotor activation by ethanol, an effect that was specific to ethanol, since other locomotor stimulating events elicited opposite responses. For example, DBA mice exhibited reduced locomotor activation in a novel environment compared to C57 mice, as shown here and by others [21]. In order to determine whether these effects originated at the dopamine terminals, we utilized fast scan cyclic voltammetry to examine dopamine release and clearance at baseline, and in the presence of ethanol, to determine if differences in these measures predict behavioral outcomes. An advantage of *ex vivo* voltammetry is that it allows for the determination of the effects of ethanol on striatal terminals, independent of afferent inputs from both the dopamine system and other neurotransmitter systems. This technique is particularly useful as many pharmacological approaches to developing treatments for psychiatric disorders rely on an understanding of the region-specific effects of drugs. Because it has been demonstrated that ethanol’s ability to increase dopamine levels is due to a balance of its actions on dopamine terminals [16] and modulation of VTA cell firing [13-15], this study determined if the actions of ethanol on dopamine terminals in the NAc core and CPu were predictive of the strain differences in the locomotor-activating effects of the drug. Our data indicate that ethanol does not change dopamine uptake, suggesting that increases in dopamine levels are via another mechanism. Further, DBA and C57 mice have similar presynaptic dopamine responses to ethanol in both striatal areas in regards to both release and dopamine uptake via the DAT, indicating that the increased sensitivity of DBA mice to the locomotor-activating effects of ethanol are likely not due to the effects of ethanol at dopamine terminals. 

## Methods

### Subjects

Male DBA and C57 mice (6 weeks old; Jackson Laboratory, Bar Harbor, ME) were used for all experiments. Animals were group housed in polycarbonate cages and maintained on a 12:12 light-dark cycle (7:00 pm lights off) with standard rodent chow and water *ad libitum*. Brain slices from both strains were obtained from naïve animals after at least one full week of habituation to the housing colony. The Institutional Animal Care and Use Committee at Wake Forest University School of Medicine approved the experimental protocol (Protocol Number: A10-177). All mice were cared for according to the National Institutes of Health guidelines in Association for Assessment and Accreditation of Laboratory Animal Care accredited facilities. 

### Locomotor Analysis

Locomotor activity was assessed via infrared beam breaks in automated locomotor activity monitors (20 cm × 20 cm × 20 cm; Med Associates, St. Albans, VT). Mice (n=9-10 per strain) were first placed into activity monitor chambers for 120 minutes to record response to novelty and to allow for habituation to the chamber. Twenty-four hours following habituation, animals were placed in the same chambers for 30 minutes before administration of two injections of saline, 60 minutes apart, to desensitize the animals to injection stress and allow for a within-subject control. Every day thereafter, animals received ethanol injections (0.125, 0.25, 0.5, 1.0, 2.0 g/kg i.p.) in the locomotor chambers after 60 minutes of habituation. Activity in response to each injection was reported for the first 30 minutes. Locomotor activity was measured as distance traveled (in centimeters) and as a percent of each animal’s second saline injection.

### In Vitro Voltammetry

Fast scan cyclic voltammetry in brain slices was used to characterize pre-drug striatal dopamine kinetics, as well as the effects of quinpirole and ethanol on single pulse evoked dopamine release. Briefly, a vibrating tissue slicer was used to prepare 400 µm thick coronal brain slices containing the striatum. Slices were incubated in oxygenated artificial cerebrospinal fluid and heated to 32°C. A carbon fiber microelectrode (≈150 µM length, 7 µM radius (Goodfellow Corporation, Berwyn, PA) and a bipolar stimulating electrode (Plastics One, Roanoke VA) were placed in close proximity (≈100 µM) on the surface of the slice, in either the NAc core or CPu. Endogenous dopamine efflux was induced by a single, rectangular, electrical pulse applied every five minutes for four milliseconds (350 µA, monophasic). Dopamine release was detected by applying a triangular waveform (-0.4 to +1.2 to -0.4 V vs. silver/silver chloride, 400 V/sec) every 100 milliseconds to the recording electrode. Background current subtraction methods were applied to obtain clear current versus time plots. When baseline collections were stable for three consecutive stimulations, quinpirole (0.01, 0.03, 0.1, 0.3, 1.0 µM; n = 7 per strain) or ethanol (25, 100, 150, 200 mM; n = 9 per strain) was bath applied cumulatively to brain slices. Electrodes were calibrated immediately following experiments by recording their response (in nA) to 3μM dopamine using a ﬂow-injection system. 

 To determine dopamine release and clearance, representative signals were analyzed before bath application of quinpirole or ethanol. Dopamine release (µM) was calculated as the amount of dopamine released per electrical stimulation whereas clearance was determined using the rate constant, tau. All data was collected and analyzed with Demon Voltammetry and Analysis software [22].

### Blood Collection and Analysis

Blood ethanol concentrations (BEC) from DBA and C57 mice were obtained at 5, 15, 30, 45, 60, 90 and 120 minutes after a 0.5 g/kg i.p. dose of ethanol (n=5-9 per strain, per time point). After injection, a submandibular vein blood draw was performed at each of the respective time points and blood was collected in BD microtainer tubes lined with lithium heparin (Becton Dickinson & Company, Franklin Lakes, NJ). Each animal had no more than two bilateral blood draws per day and blood collection volumes did not exceed the maximum set forth by the institutional Animal Care and Use Committee. For determination of BECs, standards and samples were prepared with a commercially available alcohol dehydrogenase assay (Carolina Liquid Chemistries Corporation, Brea, CA). Briefly, five microliters of each blood collection was placed in a container with 45μl of trichlorocetic acid solution (Sigma Aldrich, St. Louis, MO) and centrifuged at 10,000 revolutions per minute at room temperature for ten minutes. 30μl of the supernatant of each sample was removed and placed into a separate container with 300μl of buffer and 112.5μl of enzymatic solution provided in the alcohol dehydrogenase assay. 100μl of each standard and sample was loaded, in triplicate, into a 96 well plate, covered and incubated at 37^o^-38°C for 15 minutes. Immediately following incubation, the plate was analyzed with SoftMax Pro Software version 5 (Molecular Devices Corporation, Sunnyvale, CA). 

### Statistical Analysis

Statistical analyses and graphs were prepared using Graph Pad Prism (version 5, La Jolla, CA, USA). Summed data for the groups’ locomotor response to novelty and saline injection as well as baseline release and tau were compared across groups using a two-tailed Student’s t-test. Locomotor data (in time bins) and voltammetric data (quinpirole and ethanol concentration response curves) were compared using a two-way analysis of variance (ANOVA) with strain and dose as factors, and BECs were compared using a two-way ANOVA with strain and time as factors. When a significant main effect was obtained (p < 0.05), Bonferroni post-hoc analysis was used to determine significant effects.

## Results

### DBA mice exhibited enhanced locomotor responses to ethanol

**Figure 1:**
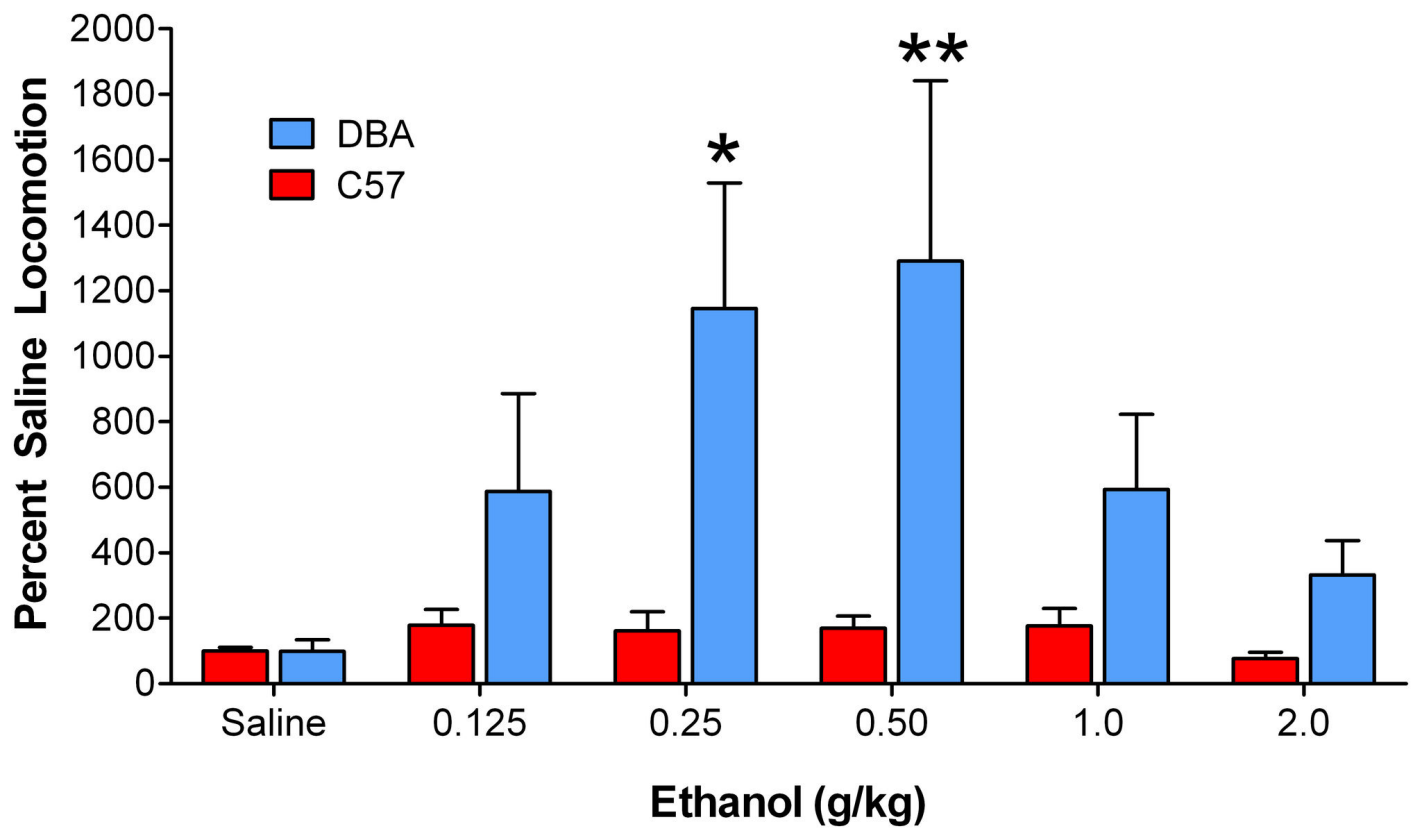
DBA mice exhibited enhanced ethanol-induced locomotor responses. DBA mice exhibited an enhanced locomotor response over a dose response curve for ethanol, as compared to C57 mice. Data is summed over the first 30 minutes post-ethanol or saline injection. *, p < 0.05; **, p < 0.01; EtOH, ethanol.

To study the behavioral sensitivity of DBA and C57 mice to ethanol, we determined ethanol-induced locomotor activity over a range of ethanol doses (0.125-2.0 g/kg). Two-way ANOVA revealed a main effect of strain on ethanol-induced locomotor behavior (F_1, 19_ = 14.68, *p* < 0.001; Figure 1). Bonferroni post hoc analysis revealed a significantly greater locomotor response of DBA mice at the 0.25 (*p* < 0.05) and 0.5 (*p* < 0.01) g/kg doses. Analysis revealed no difference between DBA and C57 mice, with respect to their locomotor response to a saline injection, indicating that basal locomotor activity did not differ between the two strains. Because DBA and C57 mice do not differ in their responses to a saline injection, their differential behavioral responses to ethanol cannot be attributed to disparate baseline locomotor activity levels. 

### DBA mice had reduced locomotor responses to a novel environment

**Figure 2:**
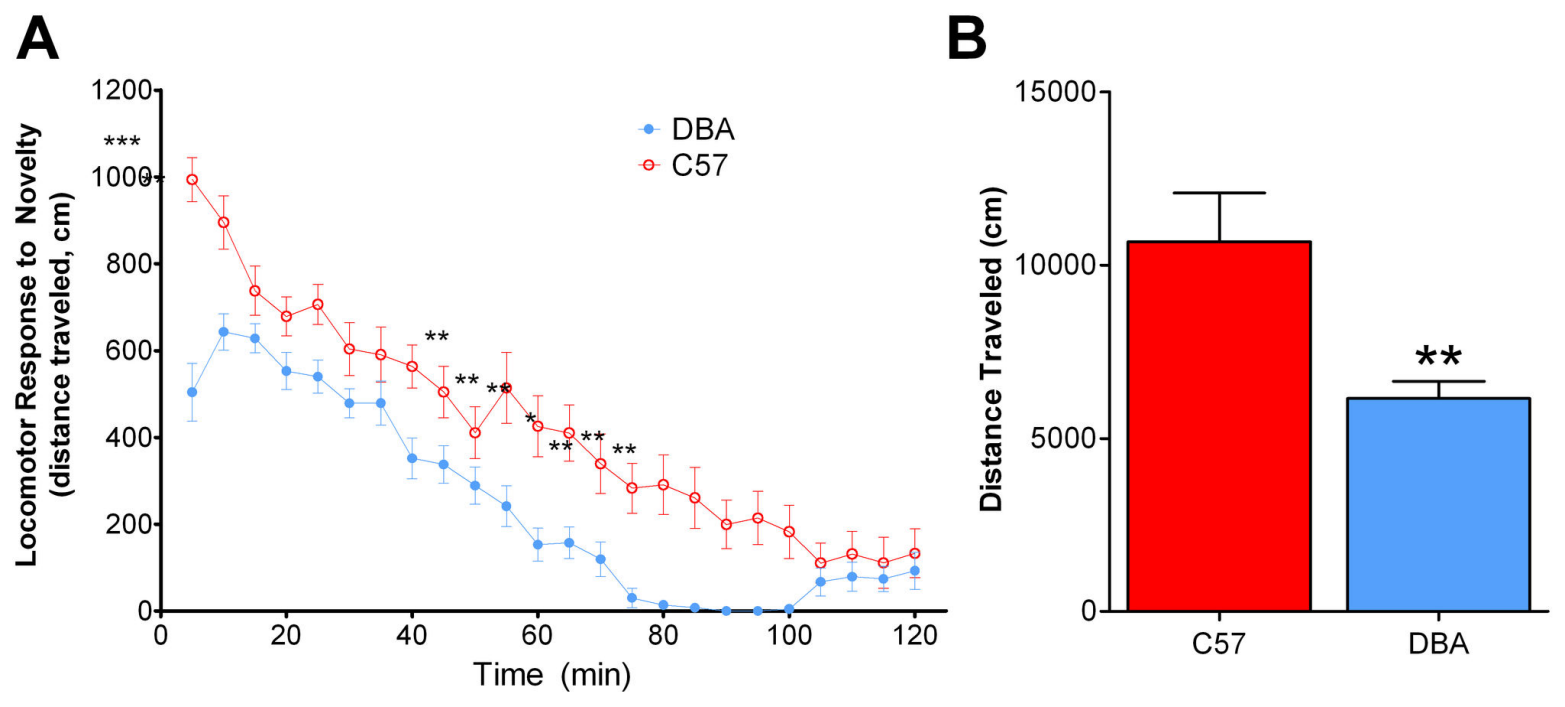
DBA mice exhibited reduced responses to a novel environment. (**A**) DBA mice showed a reduced response to a novel environment compared to C57 mice over a 120-minute locomotor session. (**B**) Summed data from the 120-minute locomotor session. *, p < 0.05; **, p < 0.01; ***, p < 0.001; min, minute.

To determine if the enhanced response to ethanol in DBA mice was specific to ethanol or was due to an enhanced response to all locomotor-activating stimuli, we examined locomotor responses to a novel environment in DBA and C57 mice. Two-way ANOVA revealed a main effect of strain on response to novelty (F_1, 39_ = 13.45, p < 0.01; Figure 2A). Bonferroni post hoc analysis revealed a reduced response to novelty in DBA mice, compared to C57 mice during the first 10 (t = 6.972, p < 0.01), 55 (t = 3.876, p < 0.01), 60 (t = 3.878, p < 0.01) 65 (t = 3.600, p < 0.01), 70 (t = 3.132, p < 0.05), 75 (t = 3.599, p < 0.01), 80 (t = 3.943, p < 0.01) and 85 (t = 3.602, p < 0.01) minute time points (Figure 2A). Summed data showed a similar trend with DBA mice exhibiting reduced novelty responding as compared to C57 mice (Figure 2B; t_18_ = 3.034, *p* < 0.01). Furthermore, we found an effect of time on locomotor response to novelty (F_23, 39_ = 87.94, p < 0.01) and a novelty x strain interaction (F_23, 39_ = 4.22, p < 0.01).

### DBA and C57 mice did not differ in blood ethanol concentrations following ethanol administration

**Figure 3:**
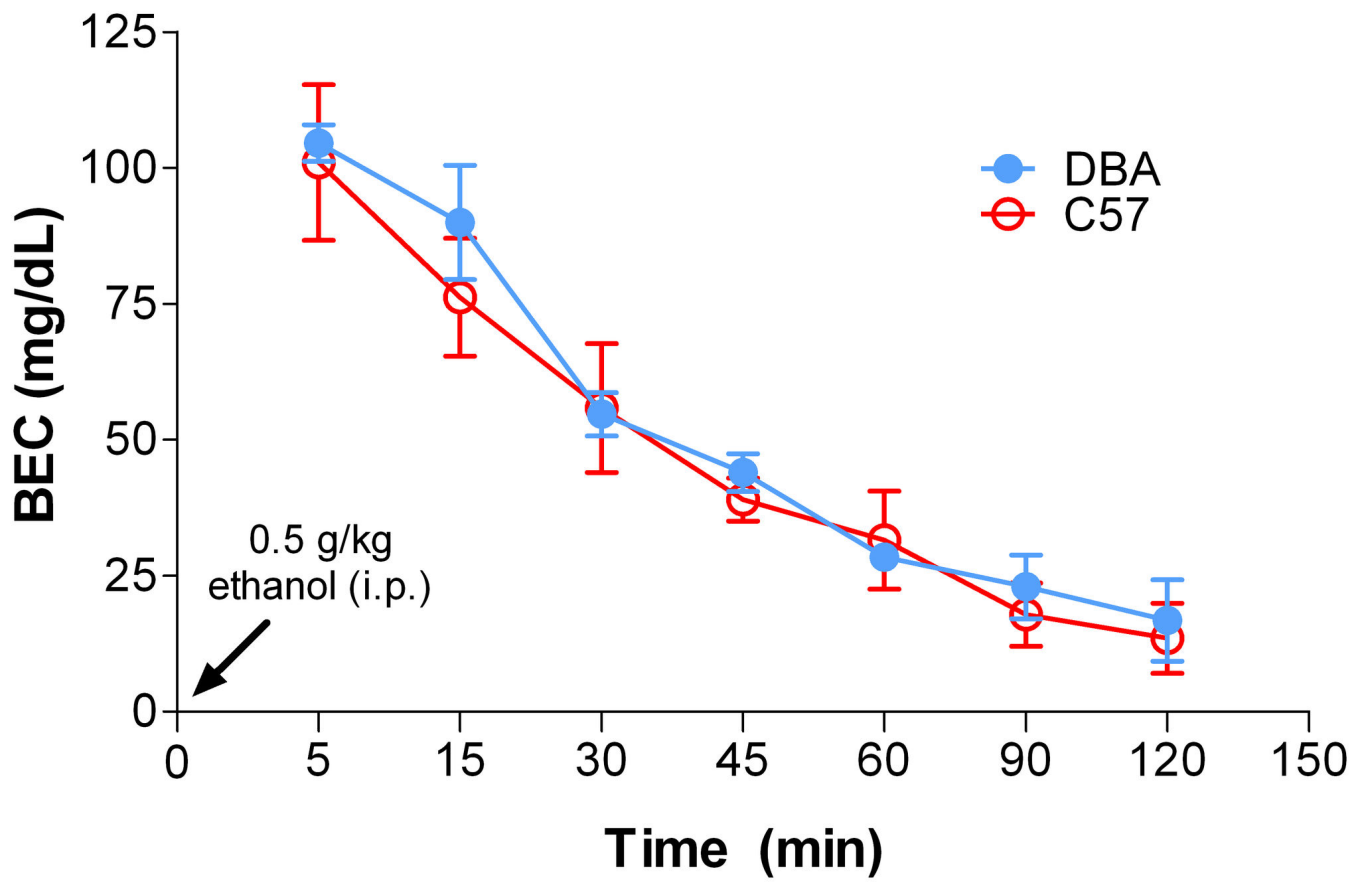
DBA and C57 mice exhibited similar ethanol elimination time courses. The time course of ethanol clearance, as measured by blood ethanol concentrations (BECs) over time, was determined in C57 and DBA mice following a 0.5 g/kg ethanol challenge. There were no significant differences in ethanol clearance between the strains. Min, minute; i.p., intraperitoneal; BEC, blood ethanol concentration.

Because it is possible that strain differences in blood ethanol elimination time could be driving differences in locomotor responses to ethanol, we examined the time course of BECs after a 0.5 g/kg i.p. injection in DBA and C57 mice. We found a significant effect of time on BECs (two-way ANOVA: F_6,20_ = 32.55, *p* < 0.001). However, we found no differences between strains with respect to blood ethanol elimination rate at any time point tested (Figure 3).

### DBA and C57 mice had similar presynaptic dopamine dynamics and autoreceptor sensitivity in the NAc core

**Figure 4:**
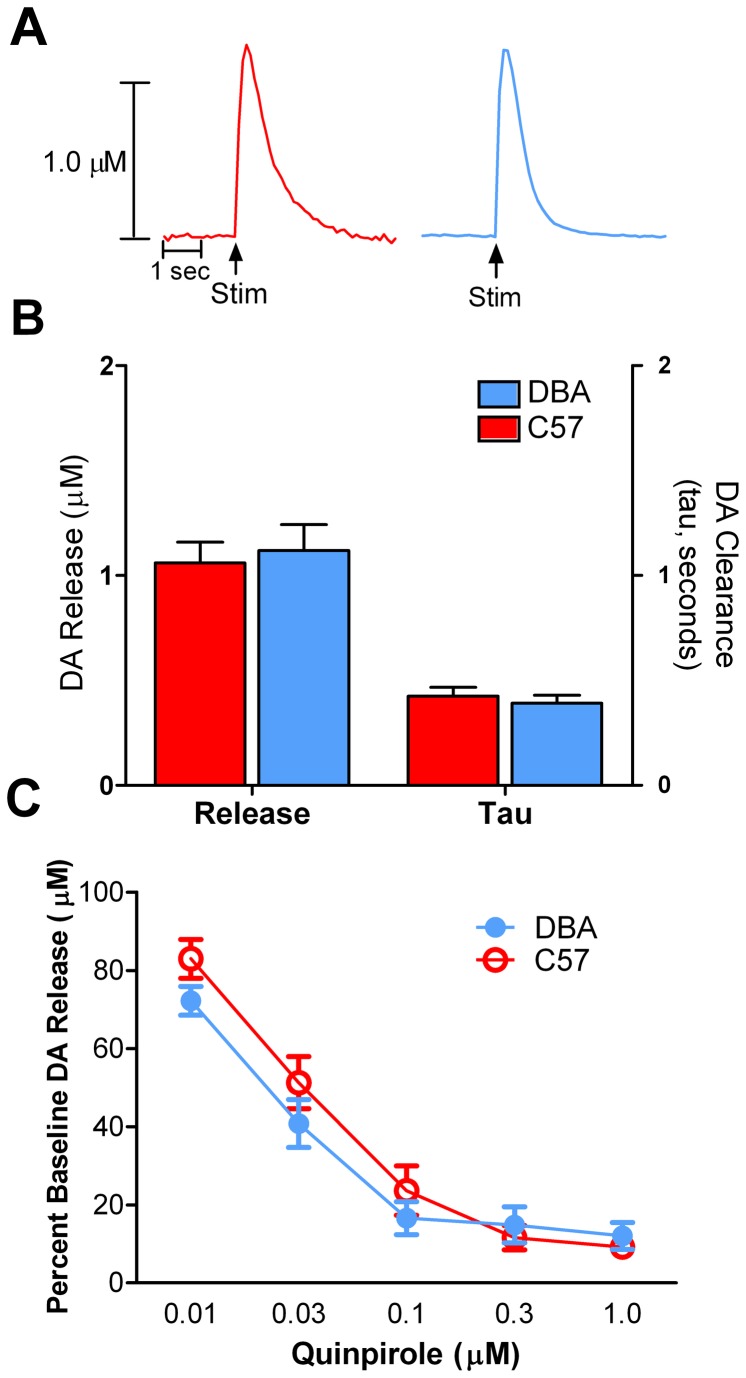
Dopamine release and clearance in the nucleus accumbens (NAc) core of DBA and C57 mice. DBA and C57 mice have similar presynaptic dopamine dynamics in the NAc core. (**A**) Raw dopamine traces from the NAc core of C57 (left; red) and DBA (right; blue) mice. (**B**) Electrically evoked dopamine release (left) and tau (dopamine clearance, right) were similar between strains. (**C**) Quinpirole, a D2-like autoreceptor agonist, was applied to brain slices containing the NAc core to determine autoreceptor sensitivity. There were no differences between strains with respect to autoreceptor sensitivity. DA, dopamine; Stim, stimulation.

Because we found robust differences in dopamine-mediated behaviors, we aimed to determine if there were differences in striatal dopamine system functioning between strains at baseline. To do this, we examined evoked dopamine release and tau, a measure of dopamine clearance, in the NAc core. We found no differences between strains in regards to dopamine release (Figure 4B, left) or clearance in this region (Figure 4B, right). 

Additionally, we assayed D2-like autoreceptor activity in the NAc core by conducting concentration-response curves for the D2/D3 agonist, quinpirole. A repeated measures two-way ANOVA revealed a significant effect of quinpirole concentration on dopamine release (F_4,19_ = 57.81, *p* < 0.001), where quinpirole dose-dependently reduced evoked dopamine release. The effects of quinpirole on dopamine release were similar between strains, demonstrating that D2-like autoreceptor function was not different (Figure 4C). 

### DBA mice have faster dopamine clearance in the CPu

**Figure 5:**
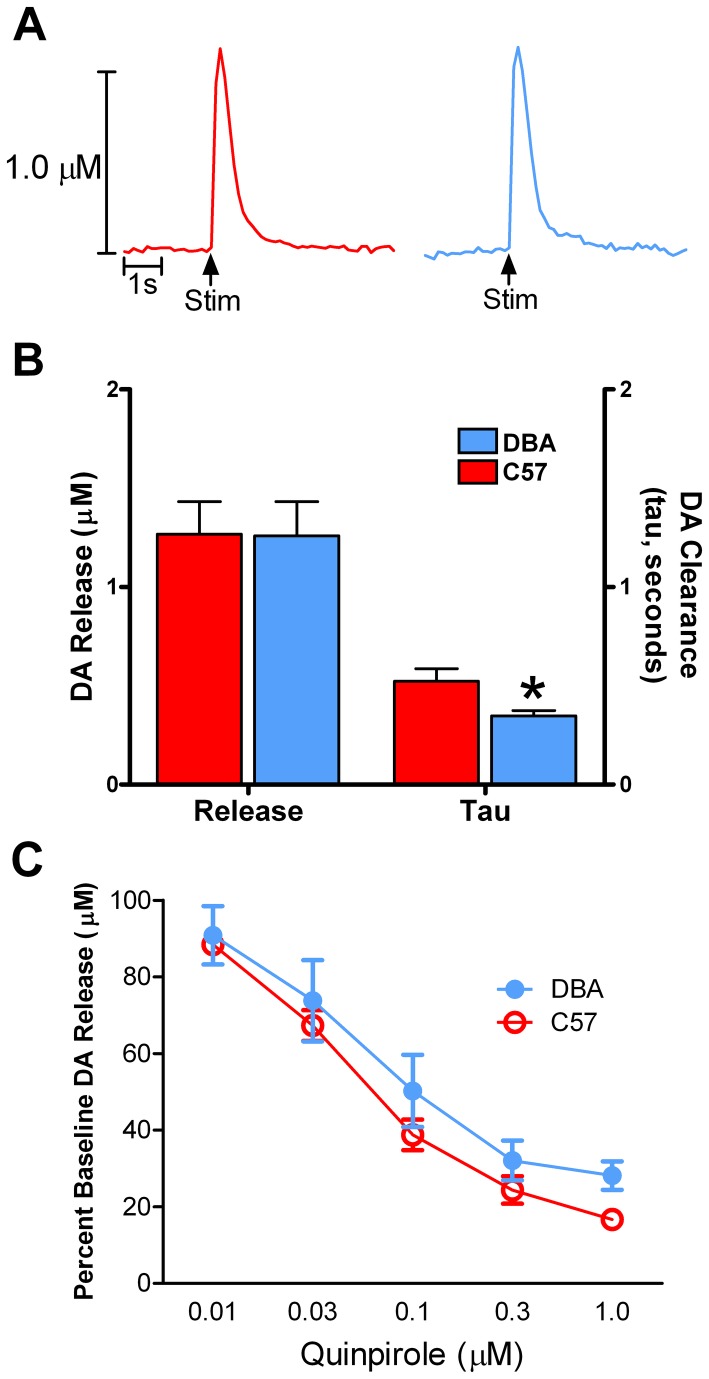
Dopamine release and clearance in the caudate-putamen (CPu) of DBA and C57 mice. (**A**) Raw dopamine traces from the CPu of C57 (left; red) and DBA (right; blue) mice. (**B**) Strains were similar in electrically evoked dopamine release (left), however DBA mice had a faster tau, indicating increased dopamine clearance (right). (**C**) The sensitivity of D2-like autoreceptors in the CPu was not different between the two strains. *, p < 0.05; DA, dopamine; Stim, stimulation.

Next, we aimed to determine if differences between strains were present in the CPu. We found that DBA and C57 mice have similar dopamine release in the CPu (Figure 5B, left); however, a Student’s t-test revealed that DBA mice had a faster rate of dopamine clearance in this region (Figure 5B, right; t_13_ = 9.43, *p* < 0.05). 

 In order to determine the D2-like autoreceptor function between the strains, we ran concentration-response curves for quinpirole. A repeated measures two-way ANOVA revealed a significant effect of quinpirole concentration on dopamine release (F_4,13_ = 41.17, *p* < 0.001), where quinpirole significantly decreased dopamine release over increasing concentrations of the compound. We found no strain differences in autoreceptor function as both strains had similar sensitivity to the effects of quinpirole (Figure 5C) in the CPu.

### The effects of ethanol on evoked dopamine release striatal subregions were not different between DBA and C57 mice

**Figure 6:**
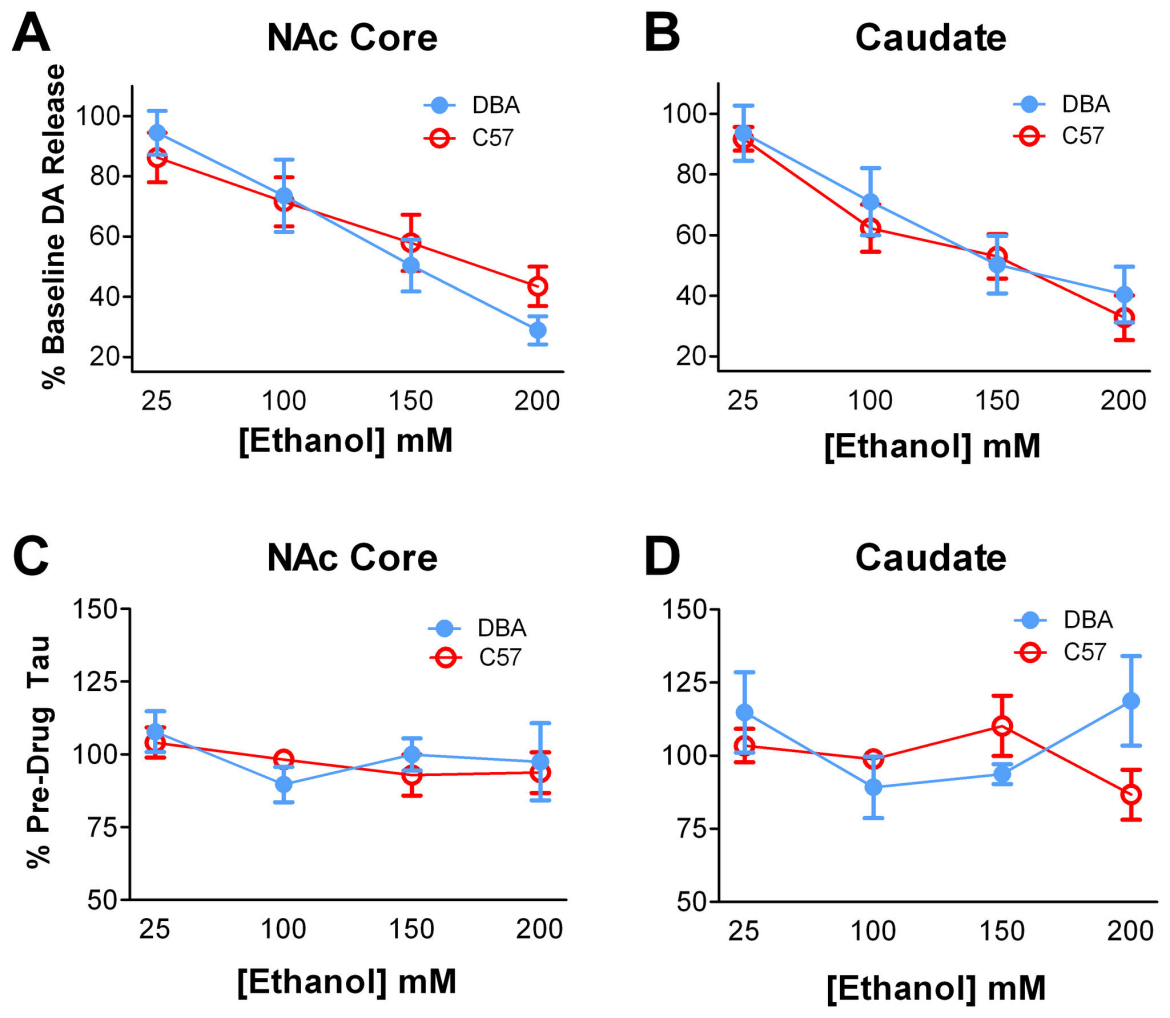
The effects of ethanol on dopamine terminals in the NAc core and CPu were similar between strains. DBA and C57 mice had similar dopamine responses to bath applied ethanol in both the NAc core (**A**) and CPu (**B**). Furthermore, brain slices from DBA and C57 mice demonstrated similar dopamine clearance rates (tau) in both the NAc core (**C**) and CPu (**D**) in the presence of increasing concentrations of ethanol. DA, dopamine.

To determine if increased ethanol-induced locomotion in DBA mice is mediated by ethanol effects on striatal dopamine terminals, we examined the effects of ethanol at the terminal by bath application of increasing doses of the drug over brain slices. A two-way repeated measures ANOVA revealed a significant effect of ethanol in both the NAc core (F_3,15_ = 14.91, *p* < 0.001) and CPu (F_3,12_ = 16.23, *p* < 0.001), where increasing concentrations of ethanol significantly reduced evoked dopamine release. We found that DBA and C57 mice have similar responses to bath-applied ethanol in both the NAc core (Figure 6A) and CPu (Figure 6B). 

In addition to determining the effects of ethanol on evoked dopamine release, we also determined the effects of ethanol on dopamine clearance. Dopamine clearance is mediated by the DAT, thus changes in the clearance of dopamine following bath application of ethanol can give information as to how ethanol alters DAT function. Contrary to previously published reports, we showed no effect of ethanol on dopamine clearance. Further, DBA and C57 mice have similar synaptic dopamine clearance measures in the NAc core (Figure 6C) and CPu (Figure 6D) in the presence of increasing concentrations of ethanol. 

## Discussion

Here we show that, although ethanol-induced locomotor activity is enhanced in DBA versus C57 mice, this difference is not due to ethanol’s effects on dopamine release or DAT activity. The ability of ethanol to reduce stimulated dopamine release in the NAc core and CPu was similar between the two strains of mice. Also, evoked dopamine release and uptake were comparable between the two strains, except in the CPu, where DBA mice exhibited faster clearance. Although some work has suggested that ethanol has direct effects at the DAT, here we show that ethanol does not influence dopamine uptake via the DAT in either the NAc core or CPu. Previous microdialysis work has shown that ethanol increases dopamine levels in the striatum to a greater extent in DBA mice [23], and this likely mediates the enhanced locomotor activity in this strain. Additionally, our data suggest that differences in ethanol-mediated increases in dopamine levels observed previously between the two strains may not be due to differential pharmacokinetic effects of ethanol, as blood ethanol elimination rates were the same. Previous work has suggested that ethanol-induced increases in dopamine levels are due to both increased dopamine cell firing in the VTA [13,14] as well as ethanol effects on striatal dopamine terminals [16]. We suggest that the elevations in dopamine levels observed in *in vivo* models following ethanol administration are most likely not due to the effects of ethanol on dopamine terminals. 

The enhanced response to ethanol in DBA mice as compared to C57 mice is not due to an enhanced response to all locomotor-activating stimuli, as DBA mice showed a reduced response to novelty. Responses to novelty have been shown previously to correlate with acquisition of stimulant self-administration and addiction vulnerability for these compounds [24-26]. Accordingly, C57 mice, which have enhanced novelty responses, are also more sensitive to the locomotor activating effects of psychostimulants [27,28]. However, while DBA mice are less sensitive to the behavioral activating effects of stimulants, they are more sensitive to ethanol, as highlighted by enhanced ethanol-induced CPP [2,3] and locomotor activity [7,8]. These data, combined with previous work, underscore the unique effects of ethanol, as DBA mice do not exhibit an increased sensitivity for all drugs of abuse. Additionally, these data suggest that there is not an overall hyperactivity of the dopamine system in DBA mice, but rather, drug-specific behavioral differences between strains. 

Although differences in locomotor activity point to differential striatal dopamine system functioning between DBA and C57 mice, our data indicate that these effects do not occur at the level of the dopamine terminal. It has been shown previously that DBA mice have enhanced ethanol-induced dopamine overflow, an effect that is likely mediating the enhanced ethanol-induced locomotion in this strain [23]. Electrophysiological reports using brain slices containing VTA dopaminergic cell bodies suggest that DBA mice have an enhanced firing rate in response to bath applied ethanol, as compared to C57 mice [13,14]. The enhanced firing could be responsible for *in vivo* increases in dopamine overflow, and could explain the locomotor differences between the two strains. In addition, postsynaptic dopamine receptors, which have been shown to have differential expression levels between the two strains, may play a role in the behavioral disparities [29]. 

Although previous research has pointed to the DAT as being altered by acute ethanol exposure [16-18], it remains uncertain as to whether ethanol increases, decreases or does not alter the function of dopamine transporters in the striatum. Here we show that ethanol does not change dopamine clearance. Voltammetric analyses of the effects of ethanol on DAT function in an *in vivo* preparation have found ethanol-induced decreases in dopamine uptake in the olfactory tubercle, an effect that could lead to increased dopamine levels following ethanol administration [16]. However, because this work was conducted *in vivo*, it is possible that ethanol effects on other neurotransmitters systems are involved in modulating dopamine dynamics, including uptake. *Ex vivo* voltammetry allows for the isolation of dopamine terminals separate from afferent inputs, which allows for the determination of ethanol effects directly at the DAT. Here we show that ethanol does not affect dopamine clearance by direct interactions with the DAT. 

 Here we demonstrate that ethanol-induced modulations of dopamine release and clearance at the level of the striatum are not mediating ethanol-induced locomotor activity. We demonstrate that the dopamine release inhibiting effects of ethanol do not differ between strains, and that ethanol does not have any direct effects at the DAT. Furthermore, our data adds to a body of literature showing that the effects of ethanol on the dopamine system are a balance of its inhibitory and excitatory effects. We show here that ethanol, when applied to the dopamine terminal, results in reduced stimulated release, while previous work has shown that ethanol, when bath applied to VTA cell bodies, results in enhanced firing. It is likely that these effects converge to result in the behavioral outputs that are observed following ethanol administration. 
